# Periscope

**Published:** 1881-06

**Authors:** 


### PERISCOPE.

*Homœopathic Journal of Obstetrics*, February.

Dr. Joslin reports several interesting cases of separation of the linea alba occurring during pregnancy and parturition.

Dr. Chapin writes upon gangrene of the mouth and vulva; gives several cases treated by swabbing with chlorate of potassa, and in the case of the vulva, "packing with camphor." There is not a word said about giving a remedy in accordance with the totality of the symptoms.

Dr. Carmichael writes upon septicæmia following abortion and contends, very justly, that it is the most frequent cause of the dangers following induced abortion. His treatment, however successful in the cases presented, can hardly be called homœopathic. Thus, on one day, in the course of treatment, he gives sulpho-carbolate of soda and quinine in alternation, and the next day he adds verat-vir. to this presciption. Then he changes to quinine, at long intervals, with veratrum and arsenic in alternation, at short intervals. Now, if this sort of treatment is successful, how does it differ from the old school? Are not these drugs used "by regular physicians" in similar conditions?

It is notorious that a crystal or two of quinia sulphate, even if added to the mucilage on the physician's desk, will preserve the paste from moulding. Here, then, is a perfectly rational reason, and an apparently unanswerable one for administering this drug in septicæmia. Why, then, should it not be used in every case and with success? What reason, then, should there be for the existence of the homœopathic method of inividualizing in such conditions? Apparently none. What need, then, have the doctors who practice in this way for calling themselves homœopathists? If they have found that there is nothing true in the creed, then they have no justification for assuming to be followers of it. Indeed, it is a duty they owe to their fellow-men to repudiate it at once, and thus assist in separating truth from error.

Dr. Wells writes an instructive paper upon "Latent Medication," in which he shows the folly of haste and foolishly changing to another remedy before the first has had time to act. This paper should be read by all true homœopathists. But those physicians who precribe remedies in alternation, who give massive doses and who use the empiricism of the old school, will not be likely to learn from it. Indeed, they will not believe any of its statements, but find in them an additional argument for the belief that Hahnemannians give no medicine at all.

Dr. Mount gives an interesting "Case in Obstetrics, contrasting the Old and New School practice." The prescriptions in this case are not up to the homœopathic mark, however.

Dr. Betts relates an interesting case of rupture of perineum in labor, which was not discovered by the medical attendant. The uterus became prolapsed and the os became firmly cicatrized into the ruptured surface. Dr. Betts discovered this state of affairs, and remedied the difficulty with a pair of scissors.

*Medical Counselor*, February and March.

Dr. Conant narrates a case of infantile colic with coryza. After unsuccessful treatment with several remedies, apparently indicated, he observed that "*the child was comparatively quiet during the day, and screamed all night.*" Jalapa 30, cured in two or three hours.

Dr. Conant also reports the following: "A lady who was recovering from a miscarriage had profuse, offensive, dark-colored uterine discharge, with back ache, prostration and procidentia uteri, the latter especially when at stool. Creosotum 6, relieved the prolapsus in three days and made a complete cure in two or three weeks.

Another lady had profuse leucorrhœa, especially when standing or walking; so corrosive that it caused swelling, soreness and itching of genitals; weakness; exertion caused profuse perspiration, trembling and shivering. Must sit near the stove. Creosote relieved in three days and cured in a few weeks.

A gentleman having paralysis of his legs had been given up to die by his allopathic adviser. He fell into Dr. Conant's hands, who prescribed *nux vom.* 1st, then 3rd, then 30th, which entirely restored his limbs to usefulness.

A farmer had floury white sediment in urine, nervous-jumping and starting at the least noise; passing of a large round worm. Cina 30, cured.

A lawyer, fond of high living and a victim of malaria and quinine, had a "mean little belly-ache" continuing all the time. Hepar 3rd, cured.

A felt-hat worker had an eruption upon his arm showing "seams, cracks and raw spots, with here and there a blister," and intense itching. Cured with rhus tox, 3 and 30th.

Dr. W. E. Leonard writes a clear, forcible paper upon gonorrhœa, with a statement of a case partially cured. It would be well if every physician could read this paper. It contains within its narrow limits more precise and correct ideas upon this disease than can be gathered from a dozen old school works. Let this article be reprinted in pamphlet form with the addition of clear indications for remedies, and it will do much good in our school. The case related was an instance of the bad effects of suppression of gonorrhœa. The patient lay in bed heavily blanketed, with *alternate chill and fever.* Dragging in right groin. *Right testicle swollen, indurated, dark, red and sensitive*, much *depression of spirits.* Pulsatilla 200. Relief in four days. Two months later there was a re-appearance of the discharge in small amount, apparently a return of the symptoms originally suppressed.

Dr. King writes upon apomorphia and advises its use in persistent vomiting. He cured two cases of vomiting with apomorphia. Indications, *vomiting after eating without previous nausea. Vomiting is sudden.*

Dr. Haines relates a case of paralysis, after fright, for which he gave remedies withou benefit. Finally, he observed the symptoms —*continued talking when awake, and changing rapidly from one subject to another. Paralysis was left sided.* Lachesis made a complete cure.

Dr. Deady gives a proving of Duboisin, made in the proper manner, with 1st, 3d and 4th decimal dilutions. Symptoms were sought for with the ophthalmoscope and laryngoscope. We quote one characteristic symptom of the eye. *Loss of accommodation before the pupil is fully dilated, and continuing when it has regained its normal size.*

Dr. Eggleston writes a condemnation of the use of the abdominal bandage in dysmenorrhœa. His argument is logical, and conclusions therefore correct.

Dr. Casseday relates five cases of severe cough cured with naphthaline 3 x. Indications: *excesssive spasmodic cough; paroxysms lasting a long time.*

Dr. Casseday cured *constant nausea with violent retching and vomiting, at short intervals, and intense headache, with* iris-vers.

*Cincinnati Medical Advance*, March.

Dr. Brigham writes a very good paper upon gynæcology, with indications for several remedies.

Dr. Smith, editor of the microscopical department, advocates an "ideal series of objectives for microscopic work."

*United States Medical Investigator*, March.

Dr. Ellis denounces the giving of "new or unchurned milk" in fevers, on account of the oil in it. He advocates instead buttermilk, because the oil has been churned out.

Dr. Morgan denounces the empiricism of the usual treatment for burns and scalds. He finds the true simillimum in cantharides, one drachm of the tincture to four ounces of water. Bathe with this dilution for half an hour or until burning pains cease.

Dr. Gilcheist points out the decline of the practice of "Listerism," or "antiseptic surgery" among the old school doctors. He claims superior results in the treatment of strangulated hernia after the operation by following the homœopathic law exclusively. Instead of administering morphia he gives hypericum. "It seems to make little difference whether the remedy is used in tincture, the 30th or the 200th attenuation."

"L" reports several cures with a new remedy—*Monotropa Uniflora.* Two cases of convulsions in children, with constipation, were cured after other remedies had failed.

Two cases of conjunctivitis — one of two years' standing— were cured, whilst a third was benefitted.

Dr. Woodbury writes upon "Reflex Gastric Derangements During Pregnancy." His most successful remedies are nux. vom., créosote and lactic acid. The last is indicated where the *nausea is constant.* He relates the case of a woman who had two miscarriages as a result of severe vomiting; but afterwards, by the help of lactic acid, had gone through three pregnancies successfully. The doctor also gives pepsin with success. He meets the objection that it is not homœopathic, with the counter objection that it is not allopathic. "It is simply supplying an element necessary to digestion." We may answer that this is not proved. Because pepsin will digest coagulated albumen of egg in a bottle kept at 99 deg. is no *proof* that it will do the same thing in the stomach. How do we know that pepsin is not attacked by the stomach juices and itself digested, just as if it were a food? Why may not this foreign pepsin act the part of a remedy—one of the so-called "nosodes"? Even the druggists, in their advertisements, are heralding the inefficiency of pepsin; especially druggists who have for sale a similar digestive substance prepared from the gizzard of the barn-yard fowl!

Dr. Pease had a horse that suffered from a bleeding wart two inches long and three quarters of an inch thick. Cured in two weeks with thuja 200 one dose.

*Bibliotheque Homœopathique*, January, February, March.

Dr. Simon reports the following cases: Catarrhal Intermittent Fever. A girl having taken cold, was seized with a severe chill followed by fever, great heat of skin, rapid full pulse, severe back ache, great thirst, tongue covered with white mucous coat, red on the edges, abdomen tense and sensitive, dry cough in short, frequent paroxysms, sibilant mucous rales all over the chest. Relieved at once by bryonia 12. The fever symptoms then returned and were treated with acon., ars., cedron 3rd, sulphate quinine, 3rd; and, finally, aranea 12th, which cured.

Phlyctenoid Erysipelas of the face, commencing on the left side and going over to the right. "Aconite as soon as the eruption was well developed: bell. followed: rhus tox given as soon as vesicles appeared; merc.-sol. after rhus; finally, sulphur to hasten desquamation."

Cystitis in a servant girl. "Sharp, burning pain whenever she has desire to urinate; continuing at the time of micturition and increasing after the act, with spasm. Bell. was given without relief. Cantharis was then given, which removed the spasms. Dulc. merc.-sol,, puls., cannab. and thuja were subsequently given in the order named. Cured. The objection to this case is the frequency with which the medicines were prescribed—not allowing sufficient time for any particular medicine to develop its effects.

Erythema of thighs and lower limbs, cured with rhus tox, followed by æsculus hipp.

Results of starvation in an Italian of 72 years. Treated with soups, wine and water, and the following remedies: Carbo. veg., puls., hepar., ars., china., cured.

Dr. Chancerel reports the following: chronic bronchitis. A tailor had a cough with hemoptysis for four years: expectoration streaked with blood. Dyspnœa, sonorous rales. Cough and dyspnœa relieved immediately by puls. 30. Symptoms became worse from too frequent repetition of the medicine. Ten days later they were much better, but he had great weakness and dyspnœa on walking. Arsenicum 30 relieved him immediately. Later, phosphorous 30 was given, which cured.

Chronic enteritis: a hack driver had chronic diarrhœa, worse from the least cold or change in diet; cold feet. Ars. 30th cured in four weeks.

V. V. writes upon "Kali Carb. in Hooping Cough." Its indications are: *cough coming on at 3 or 4 o'clock a. m., and continuing every half hour until after 5 a. m. Swelling over upper eye lid.*

Drosera has the following indications: *Cough coming on at 2 p. m., so violent that it seems as if the patient must suffocate. Pain in chest when coughing. When coughing vomiting of food, or if stomach be empty, of mucus in rolls of*

*filaments. Cough worse from heat of bed.*

Dr. Kafka gives an elaborate proving of Carlsbad Water.

Dr. Simon quotes from a Mexican journal, *La Reforma Medica.* The journal thus deplores the progress of eclecticism: "Since this sect has commenced to dismember the new school, and to forget a great part of the sage principles of the master, there are not wanting some physicians who remain faithful to the pure doctrines; who have protested against this invasion of empiricism, routine, fantasy, and polypharmacy more or less disguised in the application of a doctrine, the unity of which enchains all the parts so solidly that if we reject or contest one, we compromise all the rest. We can never approve of the homœopathic school stopping in its career; of its sacrificing the greater part of its teachings in the vain hope of winning over its antagonists; of its abandoning, without reason, the defense and the propogation of the doctrines of the master, and contenting itself with the law of similars, which is its fundamental base, but which cannot be established without the support of the other principles of the doctrine."

Dr. Charge writes upon diarrhœa, and gives an extended repertory of indications.

Dr. Chancerel translates from the Spanish a case of "popliteal sciatica" treated by Dr. Granes. Lancinating pains in right sciatic nerve. Worse from the least pressure and *the least movement.* General trembling during the paroxysms. The patient was *irritable;* wished to be alone and despaired of cure. Bryonia 2c caused immediate relief. Then inflammatory rheumatism supervened, which was controlled by rhus. tox. 2c. The case was concluded with sulph. 200.

### *New World Medical Times*, April.

In commencing a new volume, the *Homœopathic Times* changes its name. This is an honest and bold move, which we heartily commend. The prospectus says: "There will be no change in the policy of our journal; as a matter of honesty and good taste we prefer a name which will enable us to look to the vastness of the whole of medical science rather than a single law however important." In *openly* taking this eclectic position, the *New York Medical Times* exercises a freedom of medical opinion and action whlch no one can dispute, and thereby gains the right to publish and advocate any measure it deems useful or necessary. We have never objected to any one being eclectic or allopathic, or whatsoever he pleased, but we do most earnestly deprecate any attempt to pass as homœopathic any eclectic or allopathic measures. We hope other journals, now pretending to be homœopathic, will imitate *The Times*' bold candor.

Dr. Dake "reviews" Dr. Lawton's answer to the former's question at Milwaukee. By noticing every portion of Dr. Lawton's paper *except* the explanation, Dr. Dake is enabled to conclude that his question "is yet pertinent and unanswered." That explanation amounts to this: when a given quantity of a drug or of a so-called "inert" substance is triturated with another substance in large excess as vehicle, there is not only a subdivision of particles occurring, but there is also a complete separation of particles to constantly increasing distances by the interposition of the mass of the vehicle. There is, then, more room for whatever molecular motion the particles of the drug are capable of. The vehicle, on the other hand, does not acquire the same condition, because there is no other substance to be interjected between its particles to keep them asunder. Thus a small portion of charcoal, if triturated with sugar of milk as vehicle, becomes potentized. On the other hand, a small portion of sugar of milk, if triturated with charcoal as a vehicle, becomes potentized. The idea may be roughly illustrated by supposing a box full of marbles to be tilted. There is comparatively little motion. If a handful be removed, there is more room for motion. Let all be removed except a few, and then these few can make an excursion from one end of the box to the other. Let us now enter the physical laboratory. We will find here a state of affairs almost exactly parallel to this illustration. If a charge of electricity be sent through the atmosphere of a closed glass tube, it will zig-zag and delay in its passage because of the resistance. Let a portion of the air be removed and the electric current passes more readily. Remove all the air but a minute fraction — the millionth of an atmosphere—and the current spreads out as a diffused purple light of considerable brightness, and having illuminating heating and mechanical powers. That is, these few minute particles of air are competent to rotate, at great speed, the vanes of a wind-mill. Remove the air *completely* and all the phenomena *cease.* This is Prof. Crooke's "Radiant Matter." An attentive consideration of these experiments will enable us to get a clearer idea of the condition of the molecules in a potency.

Dr. Eastman, in an article upon ulcers, reports cure of an immense ulcer extending from knee to ankle and infested with maggots—after amputation had been decided upon Permanganate Potass. in local applications destroyed the maggots. Opium 3 was given because the man lay *in a stupor with stertorous breathing, involuntary stool and urine.* Later, Silicea 30 was given followed by cure.

There must be a great deal of corrupt homœopathic practice at Ward's Island, for the doctor reports that the administration of *mercurius præcipitatus rubrum* was followed by salivation to the amount of "three or four quarts a day; at the same time the ulcer became gangrenous."

Dr. Dewey gives a case of pleuro-pneumonia. Symptoms for first prescription were: *extreme restlessness, tossing about the bed, anxiety, red face, skin dry and hot, pulse full and bounding.* Aconite tinct., much improved. Bry. 3 was next given because the *pain in right mammary region was worse from motion and deep inspiration.* After five days phos. 30 was given and later phos. 2 C. Fnally, when a *chill occurred at* 10 *A. M.*, with headache, nausea, and vomiting, then heat, then sweat. Nat. mur.

30 was given, which cured the case.

Dr. Conlyn reports the cure of acute croupous pneumonia by giving first aconite, then phos. 3d.

